# Displacements of the Uterus

**Published:** 1890-12

**Authors:** 


### DISPLACEMENTS OF THE UTERUS.

(Proceedings of I. H. A. Morning Session, June 25th, 1890.)

Dr. Bell—One of the most important lessons we learn from the allopathic system of medicine is how not to do it. There is no particular objection, from a homœopathic standpoint, to mechanical supports for the uterus any more than there is to a truss in hernia, but, as a matter of fact, there are a great many mechanical objections to these supports. Dr. Hodge, who referred all or nearly all the diseases which a woman could have to displacements of the uterus, used to be a great fellow in the allopathic school, but we seldom hear about him now. Only ten years ago there was hardly to be found a correct representation of the pelvic organs. The uterus was shown upright, the rectum and vagina as incollapsible tubes, and the bladder anywhere it could be crowded in. With such radically wrong notions every woman had a displacement and pessaries could be used wholesale. By means of frozen sections the normal position of the female pelvic organs was discovered to be very different from the old idea. The allopathic authorities found that the uterus was a movable organ, with no fixed position in which it could or should be held by a mechanical support.

The day of the pessary in the old school has practically gone by, with the exception, perhaps, of the stem pessary, of which they are very much afraid.

They have also found that mere changes in position in the uterus, backward or forward, have but little bearing on the health. Aggravated cases of displacement very often have few symptoms of ill-health, while, on the other hand, severe suffering and much sickness are often present when the degree of displacement is but slight.

There is only one condition that seems to require surgical interference, and that is procidentia with a torn entrance to the pelvis and great relaxation of the parts.

Such cases often show but very few symptoms upon which to base a prescription, and to such surgical measures are probably appropriate. Very little room anywhere has been left for mechanical supports.

Dr. Hawley—Women are no more likely to be sick because they are women than men are because they are men. Women are curable by exactly the same methods as men. I pay no more attention to the mechanical symptoms than to any others. As a rule the mechanical symptoms are all hearsay; the woman only knows her womb is out of place because some doctor has told her so, and she refers all her trouble to this dreadful misplacement, which the doctor has put into her head. You must go to work and get the case just as you would any ordinary sickness, and administer your remedy without any special reference to the mechanical symptoms. A young lady of sixteen had a trick played upon her by her little brother; he pulled away a chair just as she was about to sit down upon it. She seemed shocked, but soon recovered and went to school as usual next morning. Soon her health began to fail, and a physician was called in. He heard her history, made an examination, and pronounced her case one of retroversion of the uterus. In spite of the services of seven of the most prominent allopathic physicians of New York she remained an invalid for seven years, for three of which she did not stir out of the house. They all agreed on the diagnosis; she was also under Dr. Weir Mitchell, of Philadelphia. The history of the treatment of this case is simply astonishing, to show how little help there is in the best old-school skill. She finally came into my hands. I made no manual examination of the case. I did not even feel her pulse, but I cured her.

When her menses came on I never saw in my life such suffering; that was in November. The next March that girl got up at three in the morning with her courses on, and went home a long journey without any discomfort whatever.

Dr. Stow—As a rule I have not found it necessary to use any mechanical supports whatever in these cases, but I have found them occasionally useful.

A lady called on me for treatment at the menopause. I prescribed for her for three weeks without a digital examination. I had to make one finally, and found very pronounced retroflexion, with absorption of tissue of the cervix. Sepia covered nearly all of her symptoms; she had a dragging down outside of the limbs, crossed her limbs when she sat down; pain in back, etc. I administered Sepia and also applied a stem pessary into the mouth and neck of the uterus, having first pushed the organ into position by means of an instrument introduced into the rectum. I then kept the patient in proper position for two weeks. During that time it became necessary to withdraw the stem about every fourth day, to prevent congestion. At the expiration of two weeks I found the patient much better, and in six weeks quite well. She has since become pregnant. I do not know whether she would have got well on the Sepia alone or not, but I am inclined to think not.

Dr. Reed—It seems to me that the last speaker deprived himself of the possibility of obtaining useful information by the use of the mechanical appliance. We have the pathogenesis of Sepia, and when the symptoms of the patient correspond to that pathogenesis we must use Sepia and depend upon it; so of Lilium-tigrinum, or any other drug. I come from away back, from a country of Egyptian darkness, and when I am obliged to listen to such talk as this it reminds me of the time when I was an allopath. I have a gynæcological chair in my office, and I have not used it three times in six years, and yet I make a living. I give my patient a dose of medicine, and if it is the simillimum of the case, I know by the Eternal that it will be the cure of the case. A lady came to me, not very long ago, in a desperate condition; she had a terrible bearing-down sensation, and could not go about, because, as she said, she felt that everything was coming into the world. She had to support herself with her hand. I had some homœopathic moonshine and I gave that woman a dose of that moonshine (Lilium-tig.) and in six or seven days that woman was O. K.

He was astonished to find so many homœopathic physicians using mechanical means.

The doctor who uses mechanical supports deprives himself of valuable information.

Dr. Thompson—There is only one organ in the human body as movable as the uterus. Its mobility is essential to its physiological action; without that mobility it cannot perform its use, and I do not think we should ever interfere with it by propping it in a fixed position. These cases are curable without such interference.

Two years ago I treated a young lady about twenty years old, who had been suffering a long time with severe pains. She had been sick five or six years altogether, the trouble dating from her first menstrual flow, which had been stopped by a cold taken by going into the sea to bathe while her courses were upon her. The result was the menses were stopped with intense agony.

All the indications pointed to Dulcamara; I gave her a dose, and recommended the prone position. In the course of six months she took three doses of Dulcamara. She improved right along until she developed an irritability of temper that made it almost impossible to live in the same house with her. This was cured by Chamomilla, and there has been no trouble since—in fact, she has been in perfect health.

Nothing was done for her except the administration of these two remedies and the position recommended.

I have never found it necessary, even in the case of washer-women with procidentia, who had to be about their daily tasks, to use mechanical support.

Dr. Hawley—Dr. Stow's case reminds me of what Dr. Lippe said in a similar case of retroflexion of the uterus with waste of tissue. "Unquestionably," he said, "this waste of tissue is dynamic in its cause and how can a dynamic condition be removed by mechanical means? It is simply absurd to think so."

Dr. H. C. Allen—I think Dr. Stow gave himself away pretty badly when he said he did not know whether the support or the Sepia did the work. Dr. Stow cannot tell to-day what part the Sepia took and what part the support took in the cure. There is no means of knowing, and such clinical knowledge is worthless.

Dr. Stow—One or two words in self-defense. I wish to ask this intelligent audience if I had treated that case with the remedy alone, and allowed the woman to keep about her work without any support I could have hoped to effect a cure. (*Cries of "Yes," "Certainly," from different parts of the room.*)

Dr. Kent—I should like to stand here about two hours and a half and report just such cases as Dr. Stow has reported, cured by the internal remedy alone. I used to examine my cases very frequently so that my information as to the position or malposition of the uterus of my patients used to be more extensive than it is nowadays. I simply used to keep myself posted as a matter of clinical information. I consider Dr. Stow's treatment just the same as if he had used two remedies. He does not know whether he has cured his patient or merely palliated the trouble.

Many of these cases may be made comfortable for a time by local treatment and appliances, but it is only palliation not cure.

The worst of it is, that after such treatment, it may take St. Paul himself to cure the case. It is not real homœopathic treatment, though it might be in New York.

Dr. Custis—I do not think it is well to stop examining our cases, if only for the satisfaction it affords of giving a correct diagnosis and an intelligent prognosis to our patients, both for their protection and for our protection. It is a mistake to neglect to make a digital examination. Moreover, we cannot be sure of what we have cured, unless we have made an examination on first taking the case, and the certain knowledge so gained gives stronger and better arguments to back up such a paper as we have heard here to-day. It is not fair to say we have cured a malposition because we have removed the symptoms of malposition, unless we have first made an examination and actually found out that there was a malposition there. Then we can down allopathic objectors instead of being downed by them. A good diagnosis is a great protection and a strong argument.

Dr. Wesselhœft—I agree with Dr. Custis on that point. I think it is a very important one.

I know that many of the so-called flexions and malpositions of the uterus remain and the patient gets well, and on the other hand, I know from experience that patients will come to you saying, " Doctor, I have been treated for two years by Dr. B—— for retroversion; he says I am perfectly well now in that respect, but he wants me to go to my family physician and get the constitutional symptoms cured."

Dr. Alice B. Campbell—Dr. Wesselhœft shows that there is no necessity for physical examinations. Now I do not agree with Dr. Custis at all. I have naturally greater freedom than a man in making examinations, but I do not take advantage of that freedom because I know what it means. I know they are not necessary. You cannot tell what changes of position may occur in the uterus in twenty-four hours. I do not know why the uterus should be so universally selected as the target for examinations. You don't do that with the kidneys. You can have just as much distress without the uterus being displaced at all as you can with an apparently serious displacement. If you can alleviate the distress, it makes no difference where the uterus is. If there is no suffering it is probably all right.

I do not see why we have to go over this ground every year; it seems to me we have settled it long ago on a harmonious basis. It seems like time wasted.

Dr. H. C. Allen—It is as simple as the light of the sun; the law of similars is the ground to stand upon.

Dr. W. J. Guernsey—I think with Dr. Campbell that we waste a great deal of time in going over the same ground every year.

Dr. Butler—Is it not our duty to examine our patients as thoroughly and completely as possible?

Does not Dr. Campbell make the best examination of the kidneys that their situation and their accessibility will allow? Should not every organ be examined as far as it can be?

Dr. Stow—It seems to me there is a very nice point there. Before you can make out an accurate diagnosis of an affection of the kidneys you are obliged to make chemical examinations of the urine. You do not know what you are treating unless you do; and just so I believe when a patient comes complaining of displacement of the uterus, or, of symptoms pointing to it, it is our duty before undertaking to cure the case, to find out whether it is true or not.

Dr. Brownell—I should like to ask if the influence of the clothing is not very important in the treatment of displacement?

I believe that very often such cases are incurable unless the constant displacing effect of the clothing is removed.

Dr. Hawley—This last is a very good point. I can remember when these weaknesses were unknown. My mother had nothing of the kind. She wore a belt just below the breasts, with a long skirt, all supported by the shoulders, and I believe that was the reason she did not have any of these troubles. It is impossible to be healthy and be dressed the way most women are nowadays. A woman cannot be cured who buckles belts around her waist and suspends her skirts from her hips; some organs crowded up and some crowded down. Continued strain on a muscle will invariably cause it to relax, and this continued downward pressure causes the floor of the pelvis to finally give way and the organs are left to drop down for lack of normal support. I get many cases of this kind from the allopaths who fuss with them for years without result. I have now a case pronounced by the allopaths to be a case of ovarian tumor. It is instead a case of irritable ovary and not tumor and is getting much better.

Dr. Johnstone—I am much indebted to a patent uterine supporter for a fairly round fee. About six months ago a woman, forty-three years old, came to me with agonizing bearing-down pains in the pelvis, and a great many other symptoms. I found she had been wearing a supporter to hold her in position for the last three or four years. I simply removed it, and gave her a package of placebo powders. In two weeks she was well and found to her surprise that she was better without her supporter than with it.

Dr. Stow—I believe there is such a thing as going too far in this matter of non-interference.

There is no man or woman in this audience who is more radical or a greater stickler for right than I am. I will take second place to nobody on that score.

It seems to be the direct outcome of this discussion to discard anything and everything of a surgical nature. If we do then we may as well abandon the whole bureau of surgery, for it necessarily throws out all mechanical interference with cases. Mechanical means, appliances, and aids have a field, and are perfectly legitimate in the practice of medicine.

It may be that Sepia alone would have cured my case, but I am inclined to think not. But casting that aside we have another fact that, without any homœopathic medication, without any mechanical support, pregnancy would have resolved and cured that case.

I am certain that the patient was vastly benefited, whether you attribute it to the Sepia, to the support, or to the pregnancy, and finally you must either exclude the whole subject of surgery from medicine or you must admit its technique.

Dr. Custis—I wish to set myself right with Dr. Stow. I do not want to be understood as examining every lady that comes to my office, but when we get no result from our treatment, I think we should make an examination to find out the trouble. We should feel very badly if after we had failed to make an examination and also failed to cure a case some other doctor should find a fibroid which we knew nothing about. It could not fail to be a reflection on us.

